# Hyperglycaemia and apoptosis of microglial cells in human septic shock

**DOI:** 10.1186/cc10244

**Published:** 2011-05-25

**Authors:** Andrea Polito, Jean-Philippe Brouland, Raphael Porcher, Romain Sonneville, Shidasp Siami, Robert D Stevens, Céline Guidoux, Virginie Maxime, Geoffroy Lorin de la Grandmaison, Fabrice C Chrétien, Françoise Gray, Djillali Annane, Tarek Sharshar

**Affiliations:** 1 General Intensive Care Medicine, Raymond Poincaré Hospital (AP-HP), University of Versailles Saint Quentin en Yvelines, 104 bd R. Poincaré, Garches 92210, France; 2 Department of Pathology, Lariboisière Hospital (AP-HP), University Denis Diderot-Paris 7, 2 rue Ambroise Paré, Paris 75010, France; 3 Departement of Biostatistic and Medical Informatics, Saint-Louis Hospital (APHP), University Denis Diderot-Paris 7, 47-83, boulevard de l'Hôpital, Paris 75010, France; 4 Department of Anesthesiology and Critical Care Medicine, Johns Hopkins University School of Medicine, 600 North Wolfe Street, Baltimore, MD 21287, USA; 5 Department of Pathology, Raymond Poincaré Hospital (AP-HP), University of Versailles Saint Quentin en Yvelines, 104 bd R. Poincaré, Garches 92210, France; 6 HISTO, Human Histopathology and Animal Models; Institut Pasteur; Département Infection et Epidémiologie, 25 rue du Dr Roux, 75015 Paris

## Abstract

**Introduction:**

The effect of hyperglycaemia on the brain cells of septic shock patients is unknown. The objective of this study was to evaluate the relationship between hyperglycaemia and apoptosis in the brains of septic shock patients.

**Methods:**

In a prospective study of 17 patients who died from septic shock, hippocampal tissue was assessed for neuronal ischaemia, neuronal and microglial apoptosis, neuronal Glucose Transporter (GLUT) 4, endothelial inducible Nitric Oxide Synthase (iNOS), microglial GLUT5 expression, microglial and astrocyte activation. Blood glucose (BG) was recorded five times a day from ICU admission to death. Hyperglycaemia was defined as a BG 200 mg/dL g/l and the area under the BG curve (AUBGC) > 2 g/l was assessed.

**Results:**

Median BG over ICU stay was 2.2 g/l. Neuronal apoptosis was correlated with endothelial iNOS expression (rho = 0.68, *P *= 0.04), while microglial apoptosis was associated with AUBGC > 2 g/l (rho = 0.70; *P *= 0.002). Neuronal and microglial apoptosis correlated with each other (rho = 0.69, *P *= 0.006), but neither correlated with the duration of septic shock, nor with GLUT4 and 5 expression. Neuronal apoptosis and ischaemia tended to correlate with duration of hypotension.

**Conclusions:**

In patients with septic shock, neuronal apoptosis is rather associated with iNOS expression and microglial apoptosis with hyperglycaemia, possibly because GLUT5 is not downregulated. These data provide a mechanistic basis for understanding the neuroprotective effects of glycemic control.

## Introduction

Sepsis and septic shock are associated with hyperglycaemia and peripheral insulin resistance [1,2]. Glycemic control strategies are commonly instituted as adjunctive therapeutic measures in critically ill patients, although recent studies have not consistently shown a benefit from intensive insulin therapy [3-6]. One argument supporting blood glucose control is that intensive insulin therapy is associated with a protective effect on the peripheral and central nervous system [7]. While it has been shown that intensive insulin therapy reduces the incidence of critical illness neuromyopathy [7,8] and that hyperglycaemia worsens brain injury in ischemic stroke [9-11] and head trauma [12], the effect of hyperglycaemia or insulin on sepsis related brain dysfunction is not well understood. A recent *in vitro *study showed that hyperglycaemia increased microglial vulnerability to lipopolysaccharide (LPS) mediated toxicity [13], through formation of oxidative free radicals. Interestingly, it has also been shown that experimental sepsis induces oxidative damages in the brain [14]. In a previous neuropathological study, we found that septic shock is associated with neuronal ischaemia, microglial activation and apoptosis as well as neuronal apoptosis, which was statistically correlated with endothelial expression of iNOS [15]. However, the relationships between BG and neuropathological findings have not been thoroughly assessed. The objective of the present study was to address this issue and also to assess whether hyperglycaemia is associated with neuronal or microglial apoptosis after adjustment to other pro-apoptotic factors. We also evaluated brain expression of Glucose Transporter (GLUT) proteins given their role in transmembrane glucose transport in neurons and microglial cells during stress conditions [16]. Assessment of the relationships between hyperglycaemia and neuropathological abnormalities might provide insight on the mechanisms of sepsis associated neurological and psycho-cognitive long-term consequences.

## Materials and methods

### Patients

We investigated consecutive patients who died from septic shock while receiving treatment in the ICU at Raymond Poincaré University Hospital, Garches, France [15]. Exclusion criteria were: age younger than 18 years; pregnancy; evidence of an underlying degenerative neurological disease determined clinically or on *post-mortem *examination, or any concomitant disease other than infection that might have accounted for shock and death. We obtained informed consent from the patient's closest relatives. The protocol was approved by the Comité Consultatif de Protection des Personnes se Prêtant à la Recherche Biomédicale de Saint Germain en Laye, France.

### Data collection

Demographic characteristics, pre-existing risk factors for vascular disease, and severity of illness using simplified acute physiology score II (SAPS II) [17] and sequential organ failure assessment (SOFA) [17,18] score were routinely recorded. Vital signs were recorded continuously, enabling calculation of duration of shock and cumulative time passed with a mean blood pressure of less than 60 mmHg. Standard laboratory tests and relevant microbiological data were recorded daily. All arterial and capillary BG levels measured between admission and death were collected. Hyperglycaemia and hypoglycaemia were considered when BG levels were above 2 g/l and 0.4 g/l, respectively [5,6,19]. Then, we assessed the highest and lowest BG, highest variation (Δmax) in one day in BG, mean BG, area under the BG curve (AUBGC), and AUBGC above 2 g/l (that is, hyperglycaemia). The AUBGC cut-off of 2 g/L was chosen because it reflected a compromise between the duration of hyperglycaemia and the value of blood glucose. This cut-off also allows to account for the irregular times intervals between sample collection. We also assessed the percentage of follow-up time in hypoglycaemia and in hyperglycaemia, as well as the proportion of patients who were treated with insulin and who developed hypoglycaemia and hyperglycaemia. We defined prolonged hyperglycaemia as BG values higher than 2 g/l more than 50% of follow-up time (with linear interpolation between two consecutive blood samplings). During the study period (from 1997 to 2001), no specific protocol for the management of hyperglycaemia had been implemented.

### Brain sampling

Brain samples were collected within 12 h of death. Gross examination of the brain was done after four to six weeks of formalin-fixation on coronal sections of the cerebral hemispheres and horizontal sections of the brain stem and cerebellum. Macroscopic changes were noted, and we selected the hippocampus for microscopic examination, after paraffin embedding. We decided to evaluate changes in the hippocampus as it is highly vulnerable to metabolic insults, hypoxemia and ischaemia [20,21]. Sections were stained with haematoxylin and eosin and Bodian silver impregnation combined with Luxol fast blue.

### Ischaemia, gliosis and apoptosis

Histological analysis was performed by one observer (FG) who was blinded to glycemic levels. As previously described [15,22], neurons were described as ischaemic when they presented with shrunken eosinophilic cytoplasm and pyknotic nuclei. Glial reaction (that is, gliosis) was identified as rod-shaped microglial cells and astrocytes with clear nuclei. Astrocyte and microglial activation was assessed by evaluating immunohistochemical expression of glial fibrillary acidic protein (GFAP, Dako, Glostrup, Denmark) and MHC class II antigens (HLA-DR) (Dako), CD68 (Dako). Axonal damage was assessed using immunohistochemistry for Amyloid Precursor Protein A4 (beta-APP) (MAB348, Chemicon, Lyon, France). Tissue expression of GLUT1 (a3536, Dako), GLUT3 (ab41525, Abcam, Cambridge, UK), GLUT4 (ab65976, Abcam) and GLUT5 (ab36057, Abcam), were also assessed as well as that of tumor necrosis factor α (TNFα) (Genzyme, Dako) and inducible NO synthase [23]. We previously found that sepsis-associated expression of TNFα and iNOS involve glial and endothelial cells, respectively [15]. Apoptosis was identified using a caspase 3 monoclonal antibody (Dako), and by *in-situ *end labelling (ISEL) [24] with use of ApopTag kit (Oncor, Gaithersburg, MD, USA). Intensity of neuronal ischaemia, gliosis, glial activation and apoptosis were graded between 0 and 3, as described elsewhere [15]. Expression of neuronal beta-APP, glial TNFα, endothelial iNOS, neuronal GLUT 1, GLUT3, GLUT4 and microglial GLUT5 expression were also graded from 0 to 3 [15]. Because immunostaining of GLUT3 was not satisfactory and that of GLUT1 immunostainings did not vary among patients, we did not assess their statistical correlation with blood glucose level.

### Statistical analyses

Quantitative and qualitative variables were expressed as median (interquartile range, IQR) and percentage, respectively. Association between continuous variables was assessed by non parametric Spearman correlation coefficient. Adjustment was performed by multiple linear models based on ranks, in accordance to the use of non-parametric rank correlation coefficients. Continuous and categorical variables were compared between groups of patients by Wilcoxon rank-sum test and Fisher's exact test, respectively. Values of *P *< 0.05 were considered as indicating statistical significance. All statistical analyses were performed using R 2.6.2 statistical software (The R Foundation for Statistical Computing, Vienna, Austria).

## Results

From 1997 to 2001, 17 patients who died from septic shock were included. Patient characteristics are presented in Table 1. Septic shock had a median duration of four days and was mainly secondary to pneumonia or cellulitis. Four patients had pre-existing diabetes mellitus. Median BG over ICU stay was 2.17 g/l. Episodes of hyperglycaemia were observed in all patients and hypoglycaemia occurred in five (29%) patients. Nine (53%) patients developed prolonged hyperglycaemia and six (35%) were treated with insulin (with mean BG level of 2.7 g/L (1.9 to 3.0)). Macroscopic findings were ischaemia (n = 12), haemorrhage (n = 9) and disseminated abcesses (n = 3). Oedema was observed in only one patient.

**Table 1:** Patients' characteristics

	Whole population (n = 17)
Women (%)	6 (35)
Age (years)	68 (53 to 72)
Cerebrovascular risk factors (%)	11 (65)
Diabetes (%)	4 (24)
Medical admission	11 (65)
Site of infection	
Lung only (%)	9 (53)
Abdominoperitoneal only (%)	0
Urinary tract only (%)	0
Cellulitis only (%)	5 (29)
> 1 site	5 (18)
Unknown	0
Positive culture at any site (%)	
Gram-positive only (%)	4 (24)
Gram-negative only (%)	6 (35)
Fungus only (%)	0
Mixed (%)	7 (411)
Positive blood culture (%)	4 (24)
SAPS-II at admission	43 (30 to 58)
Highest OSF score during ICU stay	4 (4 to 5)
Duration of septic shock (days)	4 (2 to 10)
Cumulative time spent with MAP < 60 mm Hg (h)	11 (4 to 25)
Lowest SAP (mm Hg)	57 (33 to 66)
Lowest PaO_2 _(kPa)	8.1 (6.1 to 9.0)
Lowest SaO_2 _(%)	85 (73 to 90)
Highest blood sodium level (mmol/L)	139 (135 to 149)
Lowest blood sodium level (mmol/L)	132 (128 to 137)
**Blood glucose level**	
Lowest BG (gr/l)	0.6 (0.3 to 1.1)
Highest BG (gr/l)	3.5 (3.3 to 5.4)
Δmax BG (gr/l)	3.4 (2.1 to 4.8)
Mean BG (gr/l)	2.2 (1.4 to 2.8)
Patients with hypoglycaemia (%)	5 (29)
Patients with hyperglycaemia (%)	15 (88)
Patients with prolonged hyperglycaemia (%)	9 (53)
Patients treated with insulin (%)	6 (35)
Neuronal apoptosis	1.0 (1.0 to 2.0)
Microglial apoptosis	1.0 (1.0 to 1.5)
GFAP expression	2.0 (2.0 to 3.0)
HLA-DR expression	1.0 (1.0 to 2.0)
CD68 expression	1.0 (0.5 to 1.5)
Glial TNF-α expression	1.0 (0 to 1.0)
iNOS expression	1.0 (1.0 to 1.0)
Neuronal GLUT4	1.5 (1.0 to 2.0)
Microglial GLUT5	1.0 (0.5 to 1.0)
CD68	1.5 (1.0 to 2.0)
Beta-APP	1.0 (1.0 to 1.5)

Beta APP, beta-amyloid precursor protein; BG, blood glucose; CD68, Cluster of Differentiation; GFAP, Glial Fibrillary Acid Protein; GLUT, glucose transporter; HLA-DR, Major Histocompatibility Complex Class II cell surface receptor; ICU, intensive care unit; iNOS, inducible Nitric Oxide Synthase; MAP, mean arterial pressure; OSF, organ systemic failure; PaO_2_, partial pressure of oxygen in arterial blood; SAP, systolic arterial pressure; SAPS-II, simplified acute physiology score; SaO_2_, saturation of oxygen in arterial blood; TNFα, tumor necrosis factor alpha.

**Figure 1:**
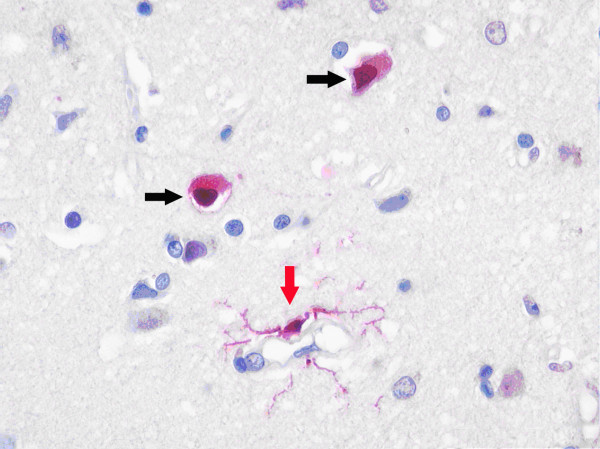
**Neuronal and microglial apoptosis in cerebral amygdale**. Case 7359, Cerebral amygdala. The back arrows show two apoptotic neurons with darkly stained nucleus. The red arrow shows an apoptotic microglial cell with a dark nucleus. The cytoplasm of the apoptotic cells is also stained corresponding to disintegration of nuclear chromatin into apoptotic bodies. (ISEL ×800).

**Figure 2:**
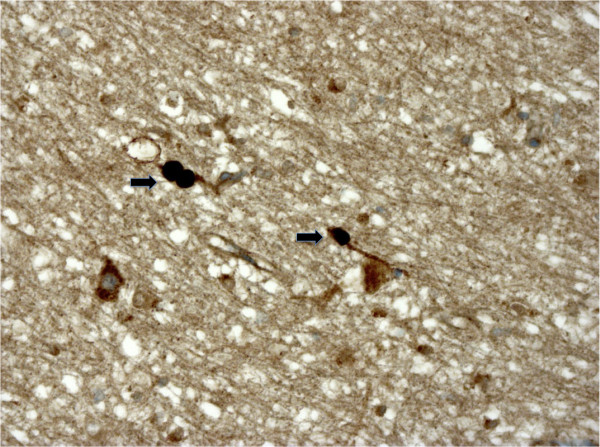
**Axonal damage in the hippocampal white matter**. Cortico-subcortical junction in the hippocampus. Black arrows show axonal swellings in the white matter. These represent the accumulation of the precursor of the beta-amyloid protein due to alteration of the axonal flow. (APP imunostaining ABC/peroxidase/DAB x25).

**Figure 3:**
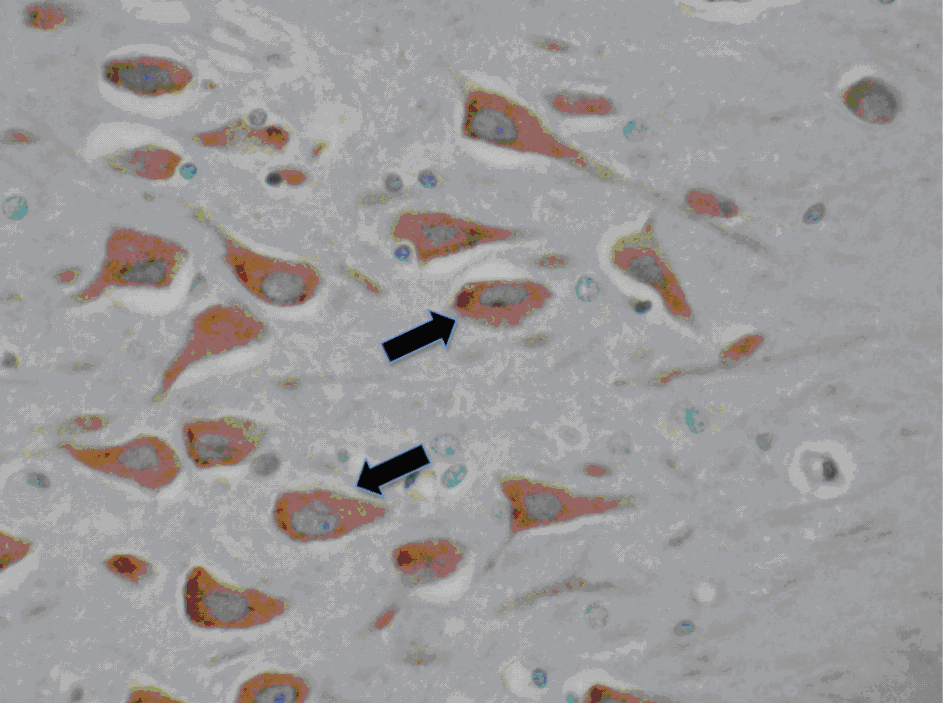
**Hippocampal expression of GLUT4**. Hippocampal interneurons in CA1 and CA4 exhibit a homogeneous cytoplasmic staining (arrow) with GLUT4 antibody (ABC/peroxidase/DAB, x40).

**Figure 4:**
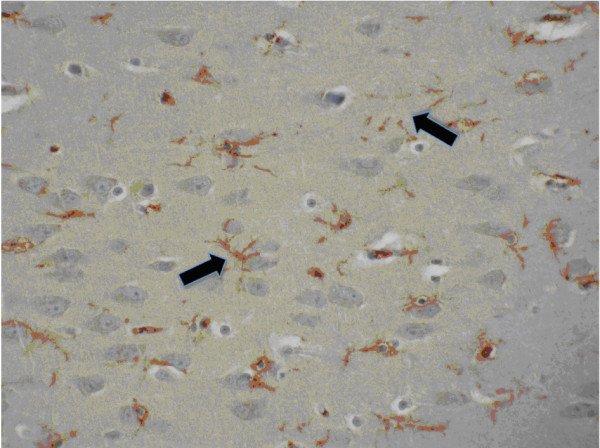
**Hippocampal expression of GLUT5**. In hippocampal interneurons (CA1 and CA4), microglial cells are strongly stained (arrows) whereas neurons are not labelled with GLUT5 antibody (ABC/peroxidase/DAB, x25).

In contrast to HLA-DR, expression of microglial CD68 tended to be correlated with AUBGC > 2 g/l (rho = 0.44, *P *= 0.08). Intensity of neuronal and microglial apoptosis was correlated with AUBGC > 2 g/l (rho = 0.53; *P *= 0.03 and rho = 0.70; *P *= 0.002) (Table 2, Figure 1). Intensity of neuronal beta-APP expression correlated with AUBGC > 2 g/l (rho = 0.61; *P *= 0.03) (Figure 2). Endothelial iNOS expression was correlated with intensity of neuronal apoptosis (rho = 0.68, *P *= 0.005) but not with that of microglial apoptosis (rho = 0.34, *P *= 0.17). The intensities of neuronal and microglial apoptosis were correlated (rho = 0.56, *P *= 0.02). Immunostaining of GLUT3 was not satisfactory. GLUT1 rather stained endothelial cells than neurons and its expression did not vary among patients. Neuronal GLUT4 (Figure 3) and microglial GLUT5 expression (Figure 4) did not correlate with prolonged hyperglycaemia nor with neuronal or microglial apoptosis (Table 3). Expressions of endothelial iNOS and microglial GLUT5 were inversely correlated (rho = -0.54; *P *= 0.03). Neuronal and microglial apoptosis were not correlated with SAPS-II at admission, highest SOFA score, duration of septic shock, or with serum sodium (especially hyponatremia), lowest systolic arterial pressure, PaO_2 _and SaO_2_. Intensity of neuronal apoptosis and ischaemia tended to be correlated with cumulative time of hypotension (rho = 0.45, *P *= 0.06 and rho = 0.38, *P *= 0.11).

**Table 2:** Association of the area under the BG curve above 2 g/l with clinical characteristics and neuropathological findings

	Spearman ρ (95%CI)	*P*
SAPS-II at admission	0.34 (-0.17 to 0.71)	0.18
Knauss	-0.21 (-0.63 to 0.30)	0.43
McCabe	0.05 (-0.44 to 0.52)	0.85
**Neuropathological findings**		
Neuronal ischaemia	0.05 (-0.43 to 0.53)	0.82
Gliosis	0.15 (-0.36 to 0.59)	0.57
GFAP expression	0.11 (-0.39 to 0.56)	0.67
HLA-DR expression	0.06 (-0.43 to 0.53)	0.81
CD68 expression	0.44 (-0.05 to 0.76)	0.08
Beta-APP expression	0.61 (0.06 to 0.88)	0.03
Neuronal apoptosis	0.53 (0.07 to 0.81)	0.028
Microglial apoptosis	0.70 (0.33 to 0.88)	0.002
Glial TNFα expression	-0.04 (-0.51 to 0.45)	0.86
Endothelial iNOS expression	0.04 (-0.45 to 0.51)	0.87

Each neuropathological finding was score from 0 to 3 (see methods). beta-APP, beta-amyloid precursor protein; CD68, Cluster of Differentiation; GFAP, glial fibrillary acidic protein; HLA-DR, Major Histocompatibility Complex Class II cell surface receptor; iNOS, inducible Nitric Oxide Synthase; TNFα, tumor necrosis factor alpha.

**Table 3:** Association of neuronal GLUT4 and microglial GLUT5 expression with glycaemia and cell apoptosis

Spearman ρ (95% CI)	Neuronal GLUT4 expression	*P*	Microglial GLUT5 expression	*P*
Area under the BG curve > 2 g/l	-0.006 (-0.49 to 0.48)	0.98	0.03 (-0.46 to 0.50)	0.91
Neuronal apoptosis	-0.29 (-0.68 to 0.22)	0.25	-0.40 (-0.74 to 0.10)	0.11
Microglial apoptosis	-0.002(-0.48 to 0.48)	0.99	-0.25 (-0.65 to 0.26)	0.33

Each neuropathological finding was score from 0 to 3 (see methods). BG, blood glucose; GLUT, glucose transporter.

## Discussion

In patients dying of septic shock, hyperglycaemia was associated with microglial apoptosis while neuronal apoptosis was preferentially associated with endothelial iNOS expression. We also found that hyperglycaemia tended to be correlated with CD68 expression, which is a marker of microglial activation. The postulated relationship between hyperglycaemia and microglial cell apoptosis was supported by its absence of statistical correlation with hypotension, hypoxemia or hypernatremia, while it is known that hippocampus is highly vulnerable to these factors. We also found that neuronal GLUT4 and microglial GLUT5 expressions were not correlated with blood glucose level, suggesting impaired downregulation.

These results are consistent with several experimental studies. Discrepancy between microglial CD68 and HLA-DR immunostaining has been previously observed [25] and was ascribed to the fact that CD68 is a better marker of activated microglia. Nitric oxide has been extensively documented as pro-apoptotic factor, notably in experimental sepsis [26-28]. In experimental models of cerebral trauma or ischaemia, hyperglycaemia has been linked to neuronal and glial cell injury through various mechanisms including mitochondrial dysfunction, oxidative stress, inflammation and excitotoxicity [29]. Although the similar mechanisms have been implicated in sepsis associated encephalopathy, the potential contribution of hyperglycaemia had not been elucidated. It was recently shown that high glucose and LPS synergistically induce microglial apoptosis by enhancing formation of oxidative free radicals [13]. Interestingly, the statistical correlation between neuronal and microglial apoptosis suggest that they are interdependent phenomenon. It is established that neuronal function and survival is intimately linked to both astroglial and microglial cells [30]. Therefore, one may speculate that hyperglycaemia induces microglial death that, synergistically with endothelial iNOS, induces neuronal apoptosis, suggesting a mechanistic sequence to account for sepsis associated brain dysfunction. This model takes into account the inflammatory [23] and metabolic (hyperglycaemia) pathways that are a major pathophysiological process and disturbance of septic shock, respectively. The correlation between hyperglycaemia and axonal beta-APP expression is consistent with that reported in experimental brain ischaemia [31]. It suggests also another scenario in which hyperglycaemia would first induce axonal injury, then secondary degeneration of microglia [31]. Interestingly, this finding proposes a new pathophysiological mechanism for the long-term cognitive decline in septic patients [32].

The present study is the first to describe the neuropathological consequences of hyperglycaemia in patients who had died from septic shock. However, our study has several limitations. First, one may argue that apoptosis was rather a *post-mortem *phenomenon. Although this possibility cannot be ruled out, we have previously shown that cell death did not correlate with time to brain sampling [15]. Second, since BG levels were not assessed continuously, it is likely that discrete hypoglycaemic or hyperglycaemic events were not detected. However, the rate of BG assessment was not different between patients with and without hyperglycaemia or prolonged hyperglycaemia. Third, it has been shown that the capillary test does not provide an accurate measurement of BG, notably overestimating it [33]. However, despite this flaw, capillary meter is used both in clinical trials and in routine for titrating insulin therapy. It has to be noted that microglial apoptosis was also correlated with median BG. Fourth, we have limited our investigation to the hippocampus as it is highly sensitive to hemodynamic, hypoxic or metabolic insults but also involved in ICU associated delirium pathophysiology [34,35]. The impact of neuronal and microglial apoptosis on hippocampal function cannot be obviously inferred from these simple neuropathological observations. It would be of interest to determine experimentally if hyperglycaemia is associated with alterations in hippocampal electrophysiological function and with cognitive impairments mediated by hippocampal structures. It has been reported that high glucose level is associated with occurrence of delirium in ICU patients [36]. Conversely, it has been shown that infusion of glucose is a memory enhancer in septic rats, suggesting that glucose tight control, or at least hypoglicaemia, may affect hippocampal functions [37].

While we have demonstrated an association between hyperglycaemia and cell death in the brains of septic shock patients, these data do not allow us to make any definitive conclusions on hyperglycaemia as a causative mechanism for cell death. Indeed, statistical correlations between ante-mortem variables and *post-mortem *findings do not prove a causal relationship. Demonstration of such a link would require a more detailed investigation of how glucose levels affect microglial cellular and molecular function and the demonstration that glycemic control reduces microglial apoptosis. Only experimental studies could reasonably address these issues. Indeed, *post-mortem *examination does not yield insight into the proximal processes that precede apoptosis in humans. This may explain that hyperglycaemia tended to be correlated with microglial activation (reflected by CD68 expression), which is prior to apoptosis. Assessment of neuropathological effect of BG control would require brain sampling in patients who had died from septic shock and who had or not been treated with insulin therapy: a task not so easily achievable. Our neuropathological samples were obtained before the widespread implementation of glycemic control with intensive insulin therapy in many critical care units. This is illustrated by the fact that insulin was administered in a small proportion of patients and was not targeted to normoglycaemia. These observations prevented us from assessing the neuropathological effect of insulin. Moreover, anticipating a neurological benefit from insulin therapy is premature. First of all, even if microglial cells play a major role in host defence of the brain, and are involved in neuroinflammatory and neurodegenerative processes, their implication in sepsis related brain dysfunction is not demonstrated [38]. It is unknown whether microglial apoptosis is an adaptive, negligible or deleterious phenomenon. Unlike the situation in neurons, interpretation of positive ISEL staining in glial and microglial cells is not straightforward. As ISEL is not absolutely specific for double-stranded DNA breaks and can also detect single-stranded breaks as observed in cell multiplication [39], positive staining may also reflect cell proliferation. On the other hand, Petito and Roberts [40] suggested that apoptotic death of reactive astrocytes might be a physiological mechanism whereby the brain removes an excess number of astrocytes that have proliferated after certain types of brain injury. This can also apply for microglia [41]. Second, cerebral glucose metabolism is highly complex and its disturbances in sepsis insufficiently elucidated. Therefore, neuronal sensitivity to hypoglycaemia and hyperglycaemia might be deeply changed in sepsis, making the effect of insulin on neuronal metabolism unpredictable. We have found that neuronal GLUT4 and microglial GLUT5 expression were neither correlated with blood glucose levels or cell apoptosis. This does not rule out that glucose transporters are involved in cell death process. For instance, it has been experimentally shown that GLUT5 is implicated in hyperglycaemia-related microglial cell death [13]. Furthermore, one may have expected that glucose transporter expression would have been inversely proportionate to blood glucose level [40,42,43]. Therefore, it is conceivable that its absence of downregulation might have increased intracellular glucose concentration and, thereby, its toxicity. We acknowledge that absence of correlation between microglial apoptosis and GLUT expression does not rule out an alteration of GLUT functioning, which in future studies could be indirectly evaluated by measuring intracellular glucose load and protein glycation. Additionally, it is biologically plausible that hypoglycaemia potentially is far more harmful for the brain than hyperglycaemia. It will be worthwhile to assess the neuropathological correlates of hypoglycaemia in patients who had died from septic shock. This would require a greater proportion of patients who had developed hypoglycaemia than that observed in the present study. It is interesting to note that iNOS has been shown to decrease cerebral GLUT1 expression [44]. One may argue that the slight GLUT1 immunostaining of neurons reflects a downregulation. Although expression of GLUT3 could not have been assessed for technical reasons, it has to be noted that alteration of GLUT3 cannot account for the relationship between hyperglycaemia and apoptosis microglial cells as it is not expressed by these cells.

The present study suggests a similar effect on microglial GLUT5 expression. Other mechanisms could be involved, especially perivascular edema that can compromise substrate and oxygen delivery. Although we have not specifically assessed this mechanism, it is established that the BBB is altered in experimental sepsis but also in septic shock patients [45].

Despite these limitations, our study suggests that hyperglycaemia may contribute to the complex web of abnormal signalling, which causes sepsis associated brain dysfunction. Future studies should investigate the mechanisms of hyperglycaemia related microglial apoptosis, particularly the impaired downregulation of GLUT, and assess the neuropathological as well as neurological effects of BG control by insulin therapy.

## Conclusions

It appears likely that hemodynamic, inflammatory and metabolic factors contribute to brain cell dysfunction and death during septic shock, and may account for sepsis associated brain dysfunction, which is associated with increased mortality [46]. More research is needed to understand the pathogenic significance of these factors and how they may be modulated to therapeutic ends.

## Key messages

• In septic shock patients microglia is strongly activated.

• Hemodynamic, inflammatory and metabolic factors contribute to brain cell dysfunction and death during septic shock.

• Hyperglycaemia is associated with microglial apoptosis while neuronal apoptosis is preferentially associated with endothelial iNOS expression.

• Hyperglycaemia may contribute to the complex web of abnormal signaling which causes sepsis associated brain dysfunction.

## Abbreviations

iNOS: inducible Nitric Oxide Synthase; AUBGC: area under the BG curve; Beta-APP: amyloid precursor protein A4; BG: blood glucose; GLUT: glucose transporter; IQR: interquartile range; ISEL: *in-situ *end labelling; LPS: lipopolysaccharide; SAPS II: Simplified Acute Physiologic Score II; SOFA: Sequential Organ Failure Assessment; TNF-α: tumor necrosis factor α.

## Competing interests

The authors declare that they have no competing interests.

## Authors' contributions

AP conceived the study, acquired data and wrote the manuscript. JPB helped in interpretation of the data and in drafting the manuscript. RP participated in the design of the study, performed the statistical analysis and helped to draft the manuscript. RS helped to draft the manuscript. SS helped in acquisition of data and revising the manuscript, while RDS also helped to revise the manuscript. CG, FC, FG, DA and VM helped to draft the manuscript. GLG helped in acquisition and interpretation of data. TS conceived the study, participated in the design of the study and helped to draft the manuscript. All the authors read and approved the final manuscript.
